# Chair Heterogeneity Index: Describing the dose heterogeneity inside the tumor volume where there is a boost volume

**DOI:** 10.1038/s41598-018-28110-9

**Published:** 2018-06-27

**Authors:** Jinming Mu, Dan Xi, Yun Ding, Wendong Gu, Qilin Li

**Affiliations:** Department of Radiation Oncology, The Third Affiliated Hospital of Soochow University, The First Peoples’ Hospital of Changzhou, Changzhou, 213003 China

## Abstract

In this report, Chair Heterogeneity Index (CHI) was introduced to assess the dose heterogeneity inside the target with a boost volume. CHI was defined by dividing (V_*Rx*_ − V_*Dl*_) by (V_*Dm*_ − V_*Dh*_): V_*Rx*_, V_*Dl*_, V_*Dm*_ and V_*Dh*_ were four points selected from the target cumulative dose volume histogram curve. The effectiveness of CHI was validated by assessing the treatment plans for nasopharyngeal cancer (NPC, 12 cases), breast cancer after breast-conserving-surgery (BC, 10 cases), and stereotactic radiosurgery after whole brain irradiation (SRS, 9 cases). Our results indicate that both CHI and HI of the target can distinguish Volumetric Modulated Arc Therapy (VMAT) from Intensity Modulated Radiation Therapy (IMRT, p < 0.05) while the mean differences in CHI (NPC 1.16, BC 1.19 and SRS 3.3) were larger than those in HI (NPC 0.03, BC 0.02 and SRS 0.02). In addition, CHI of the combination volume (the target minus the boost) were statistically higher in VMAT than IMRT in all three kinds of cancer. In conclusion, CHI was effective in assessing the dose heterogeneity inside a target containing a boost volume.

## Introduction

Simultaneously Integrated Boost technique (SIB) is a fractionation scheme for accelerated radiation therapy^1^. With the advent of intensity modulated radiation therapy (IMRT), SIB has been widely used in radiotherapy of patients with head and neck cancer^1–3^, breast cancer^4–6^, esophageal cancer^7^, and so on. SIB delivers different doses to different target volumes within a single radiotherapy fraction, which will reduce the overall treatment time and lower the expense of patients^6–8^. Additionally, SIB may be beneficial in term of increased tumor control probability due to an increased fractional dose to the tumor bed^4,9^. For example, the standard regime of radiotherapy for patients after breast conserving surgery is to irradiate the whole breast (WBI) 45–50 Gy in about 5 weeks, followed by a boost treatment to the tumor bed for additional 10–16 Gy. The entire radiation treatment is 6–7 weeks. But with SIB, the tumor bed boost will be integrated into the WBI and the overall treatment time of radiotherapy is diminished to only 5 weeks. The fractionation dose for tumor bed is also elevated with SIB.

To evaluate the dose homogeneity of radiotherapy plans, homogeneity index (HI)^2,7^ was widely applied:

1 $$HI=\frac{{D}_{2}-{D}_{98}}{{D}_{50}}$$

where D_2_, D_50_ and D_98_ represent the doses for 2%, 50% and 98% of the target volume, respectively. Meanwhile, D_2_ and D_98_ also indicate the maximum and minimum doses received by the target. Smaller HI indicates better homogeneity inside the target volume (HI ≥ 0).

**Figure 1:**
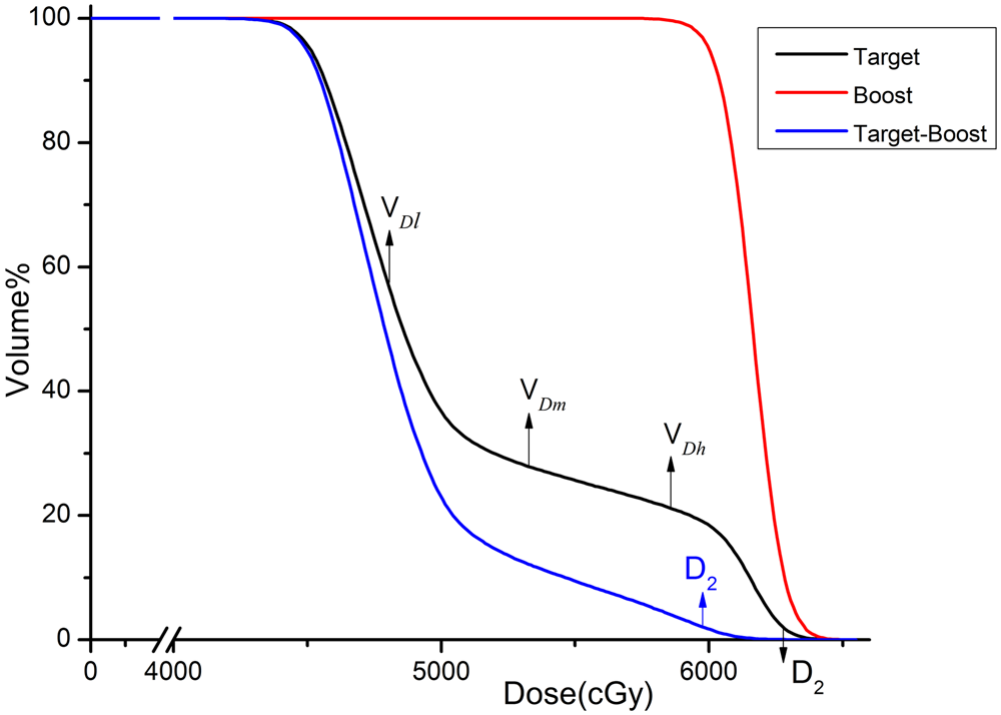
A typical dose-volume histogram of target and boost volumes. D_2_ represents the dose to 2% of the target.V_*Dl*_, V_*Dm*_, V_*Dh*_ means the percent of volume when the target receives doses of *D*_*l*_, *D*_*m*_, *D*_*h*_ respectively.

However, the application of HI in evaluating the target homogeneity for SIB would be problematic. When there is a boost volume inside the target, D_2_ is much higher than D_98_ due to the two levels of prescription dose (Fig. 1). As a consequence, Chair Heterogeneity Index (CHI) was introduced to characterize the dose homogeneity for a target volume in SIB. In this case, the ideal cumulative dose volume histogram (cDVH) curve for the target is not a vertical line but shapes like an outline of a chair (Fig. 1), which makes the CHI more suitable than HI in describing the dose heterogeneity inside the target. We further validated the effectiveness of CHI and compared it with other heterogeneity indexes.

## Materials and Methods

### Formula of chair heterogeneity index (CHI)

CHI is defined by:

2 $${\rm{CHI}}=\frac{{{\rm{V}}}_{{\rm{Rx}}}-{{\rm{V}}}_{{{\rm{D}}}_{{\rm{l}}}}}{{{\rm{V}}}_{{{\rm{D}}}_{{\rm{m}}}}-{{\rm{V}}}_{{{\rm{D}}}_{{\rm{h}}}}}$$

where D_*l*_, D_*m*_ and D_*h*_ were reference low dose, medium dose and high dose values selected between the prescription dose (Rx) of the target and of the boost (Fig. 1). Vx was the percent of the target volume received X dose. The selection for reference doses D_*l*_, D_*m*_, and D_*h*_ were specified as the following:

The chair-shaped target cumulative DVH curve could be divided into three parts (Fig. 1): the first fall-off, the plateau and the final fall-off:

1. The first fall-off was formed by the doses of the voxels inside the target but a little far from the boost—the high-dose volume. The doses of these voxels were basically free of the influence of the boost. The closer the voxel to the boost, the higher the dose to the voxel. The value of (V_Rx_ − V_D*l*_) reflects the gradient of the first fall-off of the Target cumulative DVH curve. The higher the value of (V_Rx_ − V_D*l*_) indicated the steeper the first fall-off of target cumulative DVH curve. Based on the recommendation of the ICRU Report 50^10^ that the dose coverage of the target be kept within +7% and −5% of the prescribed dose, D_*l*_ is about 7% higher than the Rx dose for the target.
2. D_m_ might be set as the mean of the Rx dose values of the main target and the boost. D_m_ should be in the plateau of the target cDVH curve. The D_m_ and D_*l*_ values so selected ensure that V_D*l*_ be greater than V_Dm_ (Fig. 1).
3. D_h_ is around 5% lower than the prescription dose to boost volume and also based on the recommendation of ICRU Report 50^10^. The value (V_*Dm*_ − V_*Dh*_) indicated the slope of the plateau of the target cDVH curve, which reflected the dose conformity around the boost. The gentler the slope of the plateau indicated better dose conformity of the boost. V_Dh_ should be less than V_Dm_.

Higher CHI, better dose distribution inside the target (outside the boost volume).

### Evaluating the SIB plans with CHI

12 patients with nasopharyngeal cancer (NPC, Stage I or II), 10 cases of whole breast irradiation with SIB after the breast conserving surgery(BC), and 9 cases of brain metastasis accepted stereotactic radiosurgery after whole brain irradiation (SRS) were selected in this study. This study was given IRB approval by the First People’s Hospital of Changzhou. Written informed consent was obtained from the patients before treatment. The methods used were in compliance with the guidelines in the Declaration of Helsinki. Patients were treated using a 6 MV photon with an Axesse^®^ linac (Elekta AB, Stockholm, Sweden). This linac is equipped with a high-definition interdigitation-capable multileaf collimator (160 leaves with a width of 5 mm at isocenter). The image guidance system is comprised of 4D cone-beam computer tomography and XVI software (version 4.5, Elekta AB), and a robotic six-degree-of -freedom patient positioning system (treatment couch HexaPOD with iGuide Software Version 1.1, Medical Intelligence, Schwabmünchen, Germany). The planning CT scan was acquired using a Siemens Somatom® Sensation Open 40-slice CT scanner (Siemens Medical Solutions, Forchheim, Germany). The slice thickness of CT images was 3 mm. The image set was exported to a Monaco Treatment Planning System (Monaco version 3.3, Elekta AB, Stockholm, Sweden) for planning. The plans were designed with two different techniques: IMRT with dynamic multileaf collimator, volumetric modulated arc therapy (VMAT). The prescription protocols for the chosen plans and the specific plan parameters were concisely listed as follows:

#### SIB plans for whole breast irradiation

1. Boost volume was generated by adding 5 mm to this tumor-bed and cropped 5 mm to the skin contour. The prescription for Boost was 60 Gy/25fractions.
2. Target volume included the glandular breast tissue cropped 5 mm inside to the skin contour. The prescription for it was 50 Gy/25fractions. Target included Boost totally.

For breast SIB plans, D_*l*_, D_*m*_, D_*h*_ in CHI were set as 53, 55 and 58 Gy respectively. The techniques for the plans: IMRT with two tangential paired fields, VMAT with a around 230° dual-arc beam (Supplemental Fig. 1). Supplemental Fig. 2 was an example of the plan constraints.

#### Plans for NPC

1. Boost volume was created with a 5 mm margin on the gross tumor volume (GTV): visible nasopharyngeal neoplasm in the images of MRI and CT, and the lymph node metastasis. The Rx dose for Boost was 70 Gy/33fractions.
2. Target included Boost and all cervical lymph drainage zones. The Rx dose for Target was 60 Gy/33 fractions.
3. For simplifying the study, the low-risk lymph nodes and lymph drainage zones were taken as a part of Target and the Rx dose was 60 Gy.

For NPC plans, D_*l*_, D_*m*_, D_*h*_ in CHI were 63, 65 and 68 Gy respectively. The techniques for the plans: IMRT with nine equidistant, coplanar fields, VMAT with one dual-arc beam.

#### Frameless stereotactic radiosurgery for brain metastases after whole brain irradiation

1. The GTV was the visible brain metastasis in the images of MRI and CT. Boost volume included GTV plus 3 mm margin. The patients enrolled had only one brain metastasis.
2. Target volume was the whole brain plus 3 mm margin.

This was a two-step plan for each patient: Target was irradiated 30 Gy/10 fractions by two parallel opposed lateral fields, and then Boost volume was irradiated 12 Gy a single treatment. The techniques of the plans for SRS: IMRT with five coplanar fields and two non-coplanar fields, VMAT with one arc beam and a partial arc beam with the couch 90° (or 270°).

**Figure 2:**
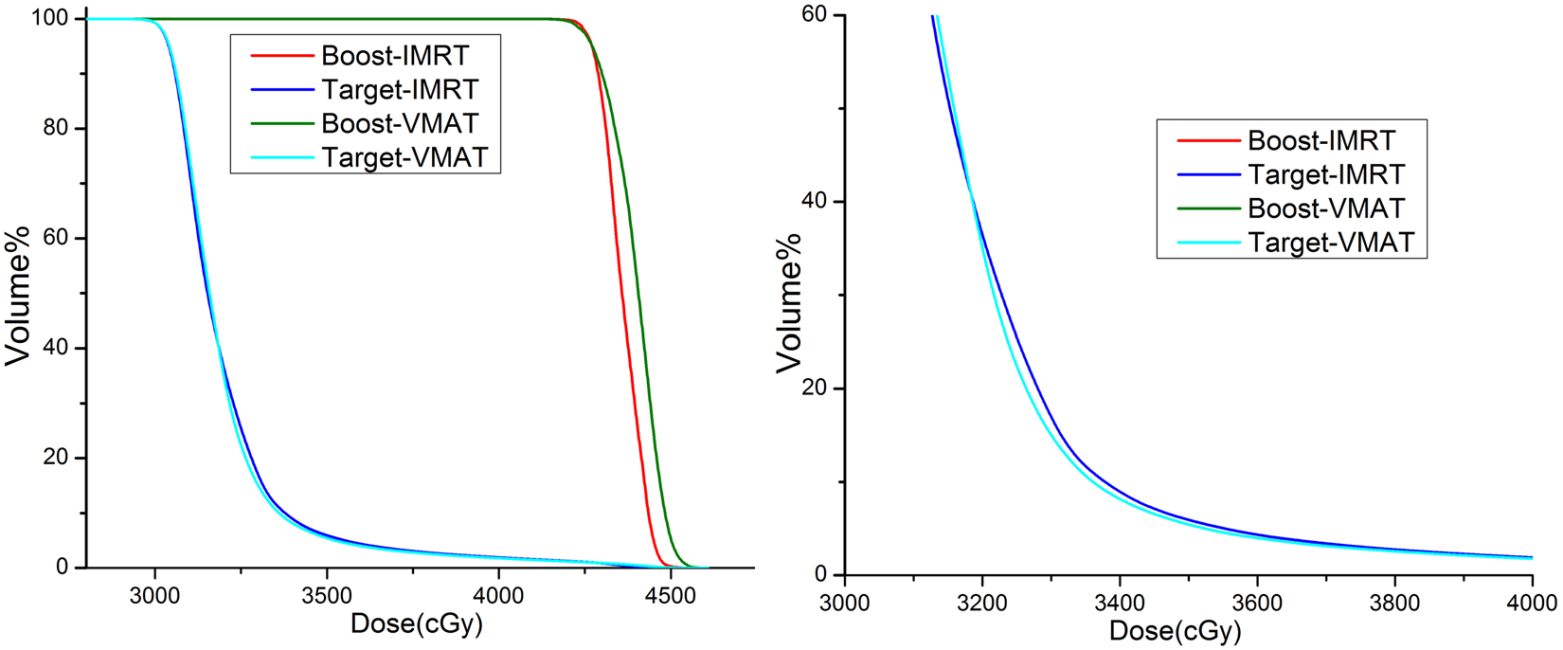
DVH of stereotactic radiotherapy for brain metastasis after whole brain irradiation. The right is an enlarged part of the DVH.

Target was irradiated first and the SRS plan for Boost was based on the dose of former plan. As a consequence, there was a gradual decrease in the first fall-off of the Target cumulative DVH curve (Fig. 2). D_*l*_, D_*m*_ and D_*h*_ in CHI were set to be 33, 36, 40 Gy respectively.

STD: Another homogeneity index^11^

3 $${STD}={{\rm{D}}}_{{\rm{SD}}}=\sqrt{\sum _{i}[{({D}_{i}-{D}_{mean})}^{2}\times \frac{{V}_{i}}{V}]}$$

where v_*i*_ is the *i*th voxel receiving a dose of *D*_*i*_, and *V* is the total volume. Here the D_*i*_ is normalized by taking the Rx dose of the target as 100. D_SD_ represents the standard deviation of the normalized voxel dose. The lower the value of STD represented the better dose homogeneity inside the target.

### Statistics

The STD, HI and CHI were calculated for each plan.The data were auto-collected with Macros from both differential and cumulative DVHs. Paired T Test and Pearson correlation test were used in this study.Two sided statistical significance level of P < 0.05 was used. All statistical analyses were performed by using SPSS software (version 13.0, SPSS Inc., Chicago, IL, USA).

## Results

Table 1 listed the relations of the volume ratio of Boost to Target (Boost/Target) with V_*Dl*_, V_*Dm*_ and V_*Dh*_. The V_*Dm*_ and V_*Dh*_ in VMAT plans have good correlations with Boost/Target, but only V_*Dh*_ in IMRT plans has statistical correlation with Boost/Target (p < 0.05). In SRS plans, all the Pearson linear correlation coefficients were greater than 0.8 between Boost/Target and the parameters V_*Dl*_, V_*Dm*_ and V_*Dh*_. The values of Boost/Target, V_*Dl*_, V_*Dm*_ and V_*Dh*_ of NPC plans were shown in Supplemental Fig. 3.

**Table 1:** The relations among the volume ratio of Boost to Target and V_*Dl*_,V_*Dm*_,V_*Dh*_ selected from the Target cumulative DVH curve (Pearson’s correlation test).

		Plan Techniques		Vs. Boost/Target			
		IMRT	VMAT	IMRT		VMAT	
		Mean ± SD	Mean ± SD	p	r	p	r
NPC	V_D*l*_	76.99 ± 5.22	65.94 ± 10.54	0.50	0.215	0.07	0.541
	V_Dm_	55.27 ± 12.17	43.83 ± 12.52	0.31	0.319	0.01	0.709
	V_Dh_	32.31 ± 11.77	27.66 ± 10.46	0.03	0.553	0.01	0.773
	Boost/Target	0.08 ± 0.04					
BC	V_D*l*_	67.14 ± 4.79	62.94 ± 4.40	0.23	−0.318	0.35	0.272
	V_Dm_	40.90 ± 6.03	33.59 ± 4.38	0.73	−0.101	0.01	0.74
	V_Dh_	22.20 ± 2.91	20.34 ± 4.14	0.00	0.815	0.00	0.995
	Boost/Target	0.15 ± 0.04					
SRS	V_D*l*_	22.02 ± 7.42	20.91 ± 8.06	0.01	0.802	0.00	0.848
	V_Dm_	5.96 ± 2.73	5.61 ± 2.54	0.00	0.896	0.00	0.894
	V_Dh_	2.43 ± 1.08	2.35 ± 1.06	0.00	0.953	0.00	0.949
	Boost/Target	0.013 ± 0.006					

Abbreviation: NPC = nasopharyngeal cancer, SRS = stereotactic radiosurgery after whole brain irradiation, BC = breast cancer.

**Figure 3:**
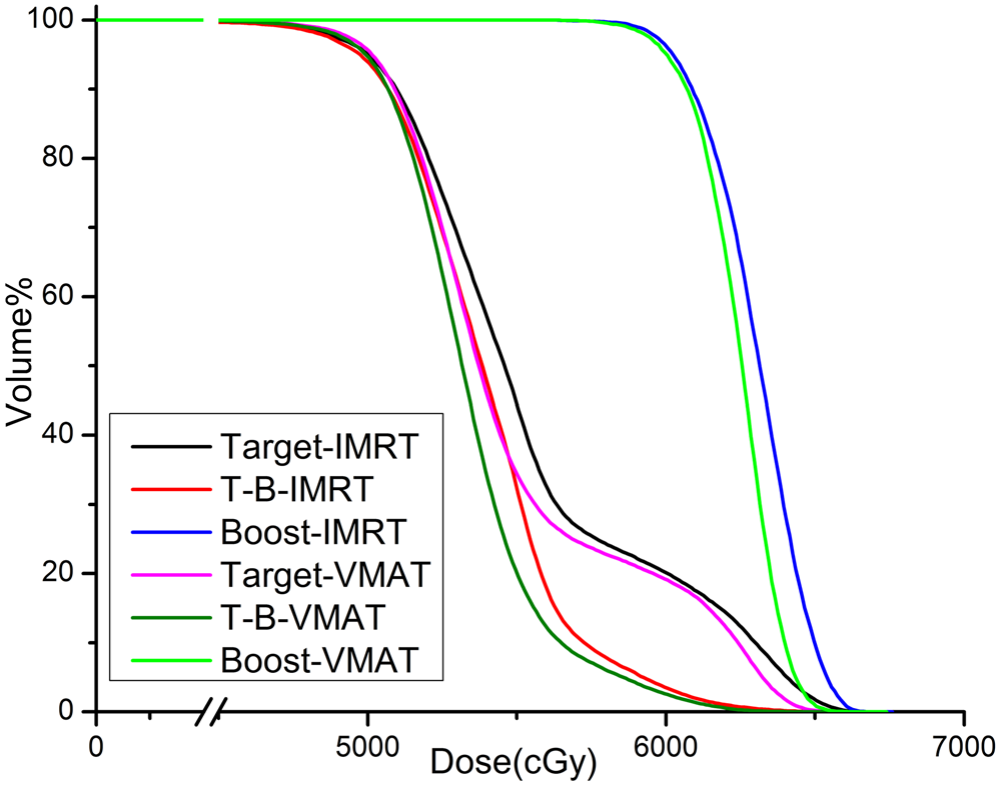
The DVH comparison between two kinds of plans on target and boost (BC).

**Figure 4:**
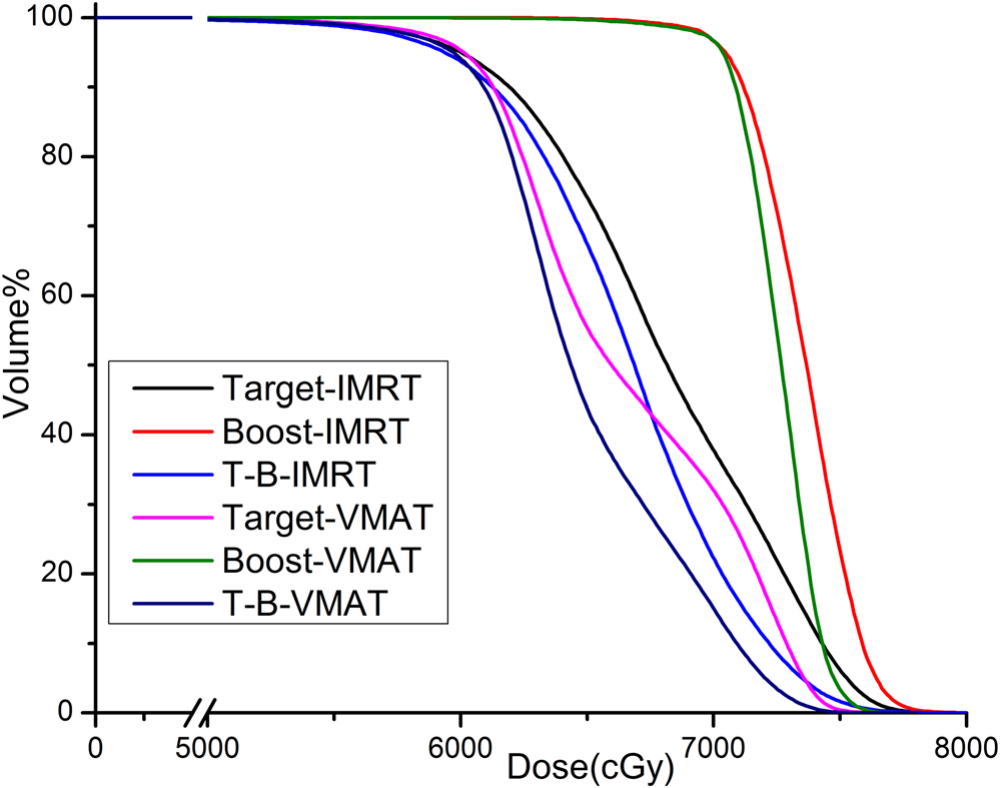
The DVH comparison between two kinds of plans on target and boost (NPC).

**Figure 5:**
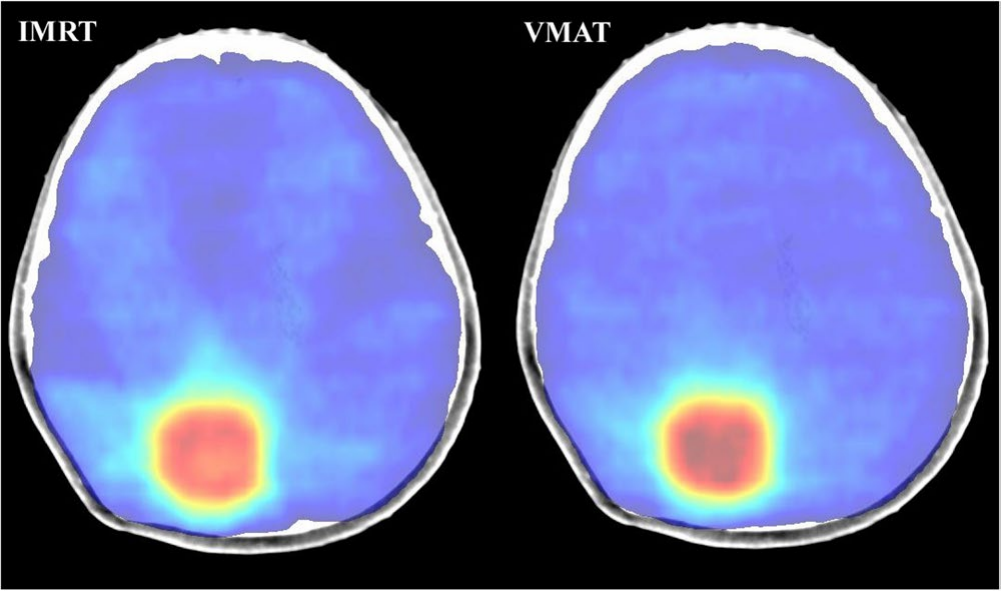
The dose distributions in one selected transverse plane. Left: IMRT technique, Right: VMAT technique, dose colorwash range: 3000–4600 cGy.

CHI, HI and STD were listed in the Table 2 for NPC plans, BC plans and SRS plans. CHI of the Targets in VMAT plans (NPC, 2.49 ± 1.08, Breast cancer, 3.51 ± 0.9 and SRS, 40.1 ± 12.2) are statistically higher than those in IMRT plans (NPC, 1.33 ± 0.58, Breast cancer, 2.32 ± 1.1 and SRS, 36.2 ± 13.6). This was also clearly shown in Figs 2–5: the Target cDVH curves of VMAT plans were better than those of IMRT plans. It was interesting that HI of the Targets in VMAT plans, like CHI, were better than those in IMRT plans for all three cancers. But the mean differences of HIs were tiny between two kinds of plans (less than 0.03 in NPC, BC and SRS plans). Unfortunately, STD of Target was failed in distinguishing two techniques in NPC as the P value is higher than 0.05 (Table 2).

**Table 2:** Three kinds of indexes in two kinds of plans for nasopharyngeal cancer, breast cancer and stereotactic radiosurgery after wholebrainirradiation.

	Tissue	Index	IMRT	VMAT	P
			Mean ± SD	Mean ± SD	
NPC	Target	CHI	0.82 ± 0.31	2.16 ± 1.21	0.002
		HI	0.28 ± 0.02	0.25 ± 0.03	0.023
		STD^+^	7.24 ± 1.00	6.92 ± 1.06	0.092
	T-B*	CHI	0.83 ± 0.31	2.17 ± 1.20	0.002
		HI	0.25 ± 0.03	0.23 ± 0.03	0.020
		STD^+^	6.12 ± 0.85	5.76 ± 0.81	0.052
	Boost	HI(%)	0.09 ± 0.02	0.08 ± 0.02	0.009
		STD^+^	2.28 ± 0.52	1.89 ± 0.46	0.010
Breast Cancer	Target	CHI	1.71 ± 0.83	2.61 ± 0.90	0.004
		HI	0.32 ± 0.02	0.30 ± 0.01	0.001
		STD^+^	8.37 ± 0.52	8.00 ± 0.56	0.017
	T-B*	CHI	1.72 ± 0.83	2.62 ± 0.90	0.004
		HI	0.25 ± 0.02	0.24 ± 0.01	0.001
		STD^+^	5.80 ± 0.48	5.33 ± 0.23	0.001
	Boost	HI	0.11 ± 0.02	0.09 ± 0.01	0.003
		STD^+^	2.83 ± 0.49	2.26 ± 0.13	0.001
SRS	Target	CHI	36.20 ± 13.61	40.08 ± 12.23	0.043
		HI	0.36 ± 0.07	0.35 ± 0.08	0.043
		STD^+^	7.46 ± 1.16	7.70 ± 1.46	0.10
	T-B*	CHI	36.20 ± 13.61	40.08 ± 12.23	0.043
		HI	0.28 ± 0.05	0.26 ± 0.05	0.020
		STD^+^	6.10 ± 0.88	5.76 ± 0.88	0.005
	Boost	HI	0.06 ± 0.01	0.08 ± 0.01	0.001
		STD^+^	1.63 ± 0.29	2.21 ± 0.32	0.002

*T-B: the combination structure of Target minus boost.

STD: the standard deviation of the normalized differential DVH curve^10^.

The statistical analyses of CHI and HI for the combination structure of T-B (Target minus Boost) were similar with those for Targets. The average CHI in T-B was very close to those in Targets, no matter what type of the cancer or the technique. In all three cancers, the differences between them were not greater than 0.01 in both VMAT and IMRT in all three cancers (Table 2).

There were only HI and STD calculated for Boost in three cancers. Table 2 indicated that both indexes are better in VMAT than in IMRT in plans for BC and NPC, but worse in VMAT than IMRT in plans for SRS.

## Discussion

There were different target volumes in Radiation Oncology which were clearly defined in The International Commission on Radiation Units and Measurements (ICRU) Report 50^10^, such as gross tumor volume (GTV), Clinical Target Volume (CTV), planning tumor volume (PTV) and so on. Subclinical microscopic extensions of tumor (SMET) were classified as one kind of CTV. As the hypoxic cancer cells may be less likely in SMET than in bulky tumors (the GTV), SMET should be controlled by a lower dose than is required for GTV^12^. In this study, Target was the low-dose volume and Boost was the high-dose volume. Target contained the entire Boost.

IMRT, including volumetric modulated arc therapy (VMAT), has the ability to deliver different doses to different volumes at the same time^12^. This feature makes the implementation of SIB feasible and simple. It has been proven that SIB is better than sequential boost in head and neck cancer, breast cancer, etc^2,3,7,9,13,14^.

However, owing to the existence of the Boost—high dose volume, we usually overlooked the dose distribution inside the combination structure (Target minus Boost) when evaluating the plan quality of SIB plans.

HI represents the gradient of the target cumulative DVH curve and is a very good index in describing the dose homogeneity in a target volume. However in case there are two prescribed dose levels HI becomes problematic. The high dose tail in the lower dose target elevates the HI value, and insensitive to the qualitative difference in the dose distribution (Table 2 and Fig. 1). For example, Table 2 indicated that VMAT is better than IMRT in the dose homogeneity inside the Boost volume in NPC and BC. The HI values of Boost were around 0.1. But the HI values of Target, the low dose volume which contained the high dose volume Boost, were dramatically up to around 0.25 and higher. The high HI values revealed that the HI may be problematic to assess the dose homogeneity in this case. The HI values of Targets in VMAT technique were statistically better than that in IMRT technique in both NPC and BC, but it might be influenced by the high dose volume (Boost) inside Target.

On the other hand, CHI was designed for this type of target. (V_*Dl*_ − V_*Dm*_) and (V_*Dm*_ − V_*Dh*_) represented the gradients of the first fall-off and the plateau of the target cumulative DVH curve, respectively. The higher the (V_*Dl*_ − V_*Dm*_) and the lower the (V_*Dm*_ − V_*Dh*_) indicated the better dose distribution inside the target. This also leaded to a high value of CHI.

As shown in the Table 2 and Supplemental Fig. 4, CHI of the Targets in VMAT were significantly higher than that in IMRT and their mean differences were obvious(1.16 for NPC and 1.19 for Breast cancer). On the contrary, for HI, the mean differences between VMAT and IMRT plans were tiny (NPC: 0.03, BC: 0.02). In conclusion, CHI was more sensitive to HI in assessing the dose distribution inside the target containing a boost volume.

In this study we also assessed the dose distribution of plans for SRS for brain metastasis after whole brain irradiation. This type of plans belonged to sequential boost technique. The characteristic of this plan is the rather small volume ratio of Boost to Target (around 1%, Table 1). This was the reason why we chose these patients to verify the efficacy of CHI. It was shown in Table 2 that HI of all volumes, including Target, T-B and Boost, were better in IMRT than in VMAT. But this did not mean that IMRT was superior to VMAT in this condition. CHIs of both Target and T-B were statistically higher in VMAT than in IMRT. And it was obviously shown in Fig. 5 that the dose distribution in VMAT is better than IMRT. It was truth that the dose homogeneity inside Boost in IMRT is better than that in VMAT. However, it was expected that there is a higher dose inside the target volume in SRS^15^. Here was the conclusion that VMAT is better than IMRT in SRS and CHI is more suitable than HI in evaluated the dose distribution inside the target with a boost volume.

We must choose D_*l*_, D_*m*_ and D_*h*_ properly and carefully, especially D_*l*_. For example in SRS, the difference of the Target cDVH curve between VMAT and IMRT was apparent at the dose range 33–35 Gy (Fig. 2). If D_*l*_ was selected in other values, we might get a wrong result. The selection of D_*m*_ and D_*h*_ was relatively easy as D_*m*_ and D_*h*_ were in the middle or the end of the plateau of the curve.

STD was the standard deviation of the Target differential DVH curve^11^ and used to assess the dose homogeneity inside the target volumes in this study. Table 2 indicated that STDs of Target and T-B were better in VMAT than those in IMRT for NPC, but the result of the paired T test showed that the differences between two techniques were insignificant at two-tailed significant level 0.05. On the other side, both CHI and STD of Target and T-B were statistically better in VMAT than those in IMRT for SRS (Table 2). The results aforementioned illustrated that STD was not superior to HI and CHI in evaluating the dose distribution inside a volume.

CHI and HI were defined with limited number of points in the Target curve of cDVH. The simplicity of the formula for HI was essential and made it attractive for people to quantify the dose homogeneity inside a volume^11^. We would like to propose that CHI will also be widely used in the near future. CHI was designed for assessing the dose distribution inside a target containing a boost volume. With the widespread use of SIB technique in the treatment for cancers, CHI is expected to be adopted as an index to evaluate the plan quality, along with the HI and conformity index.

## Electronic supplementary material

Supplemental Figure 1

Supplemental Figure 2

Supplemental Figure 3

Supplemental Figure 4
