# Supplementary material for: Chair Heterogeneity Index: Describing the dose heterogeneity inside the tumor volume where there is a boost volume
Source: Sci Rep. 2018 Jun 27;8:9763. doi: 10.1038/s41598-018-28110-9 (PMC6021440; doi:10.1038/s41598-018-28110-9)
Supplement: Supplementary file 1 — Supplemental Figure 1 [file 41598_2018_28110_MOESM1_ESM.pdf]

Chair Heterogeneity Index: Describing the dose heterogeneity inside the tumor volume where there is a boost volume

Jinming Mu* Dan Xi * Yun Ding Wendong Gu Qilin Li

Department of Radiation Oncology，The Third Affiliated Hospital of Soochow University，The First Peoples' Hospital of Changzhou，Changzhou 213003，China

** Two authors contributed equally to this work*

Corresponding authors: Mr. Qilin Li，Email: forster3602@aliyun.com

Structure	Cost Function	Enabled	Status	Manual	Weight	Reference Dose (cGy)
Boost	Target Penalty	☑	On	☐	1.00	
	Quadratic Overdose	☑	On	☐	0.01	6300.0
	Quadratic Underdose	☑	On	☐	0.01	5950.0
Target	Target Penalty	☑	On	☐	1.00	
	Quadratic Overdose	☑	On	☐	0.01	5250.0
	Quadratic Underdose	☑	On	☐	0.01	4950.0
	Quadratic Overdose	☑	On	☐	2.24	5500.0
Lung_L	Overdose DVH	☑	On	☐	24.48	500.0
	Overdose DVH	☑	On	☐	0.21	1000.0
	Overdose DVH	☑	On	☐	0.01	2000.0
	Maximum Dose	☑	On	☐	0.01	
	Quadratic Overdose	☑	On	☐	0.01	5500.0
Lung_R	Overdose DVH	☑	On	☐	0.01	500.0
SpinalCord	Maximum Dose	☑	On	☐	0.01	
Breast_R	Maximum Dose	☑	On	☐	0.01	
Heart	Overdose DVH	☑	On	☐	0.01	3000.0
	Overdose DVH	☑	On	☐	1.81	500.0

Supplemental Figure 1: Part of SIB plan constraints for the whole breast irradiation.
