# Supplementary material for: Chair Heterogeneity Index: Describing the dose heterogeneity inside the tumor volume where there is a boost volume
Source: Sci Rep. 2018 Jun 27;8:9763. doi: 10.1038/s41598-018-28110-9 (PMC6021440; doi:10.1038/s41598-018-28110-9)
Supplement: Supplementary file 2 — Supplemental Figure 2 [file 41598_2018_28110_MOESM2_ESM.pdf]

Chair Heterogeneity Index: Describing the dose heterogeneity inside the tumor volume where there is a boost volume

Jinming Mu* Dan Xi * Yun Ding Wendong Gu Qilin Li

Department of Radiation Oncology，The Third Affiliated Hospital of Soochow University，The First Peoples' Hospital of Changzhou，Changzhou 213003，China

** Two authors contributed equally to this work*

Corresponding authors: Mr. Qilin Li，Email: forster3602@aliyun.com

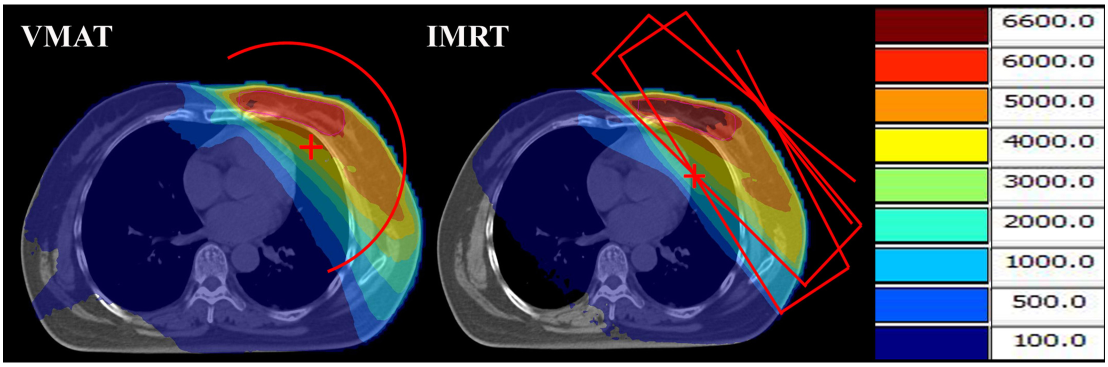

Supplemental Figure 2: The beam arrangements in SIB plan for the whole breast irradiation and the dose distributions in one selected transverse plane.
