# Supplementary material for: Chair Heterogeneity Index: Describing the dose heterogeneity inside the tumor volume where there is a boost volume
Source: Sci Rep. 2018 Jun 27;8:9763. doi: 10.1038/s41598-018-28110-9 (PMC6021440; doi:10.1038/s41598-018-28110-9)
Supplement: Supplementary file 4 — Supplemental Figure 4 [file 41598_2018_28110_MOESM4_ESM.pdf]

Chair Heterogeneity Index: Describing the dose heterogeneity inside the tumor volume where there is a boost volume

Jinming Mu*Dan Xi * Yun Ding Wendong Gu Qilin Li

Department of Radiation Oncology, The Third Affiliated Hospital of Soochow University，The First Peoples' Hospital of Changzhou，Changzhou 213003，China

** Two authors contributed equally to this work*

Corresponding authors: Mr. Qilin Li，Email: forster3602@aliyun.com

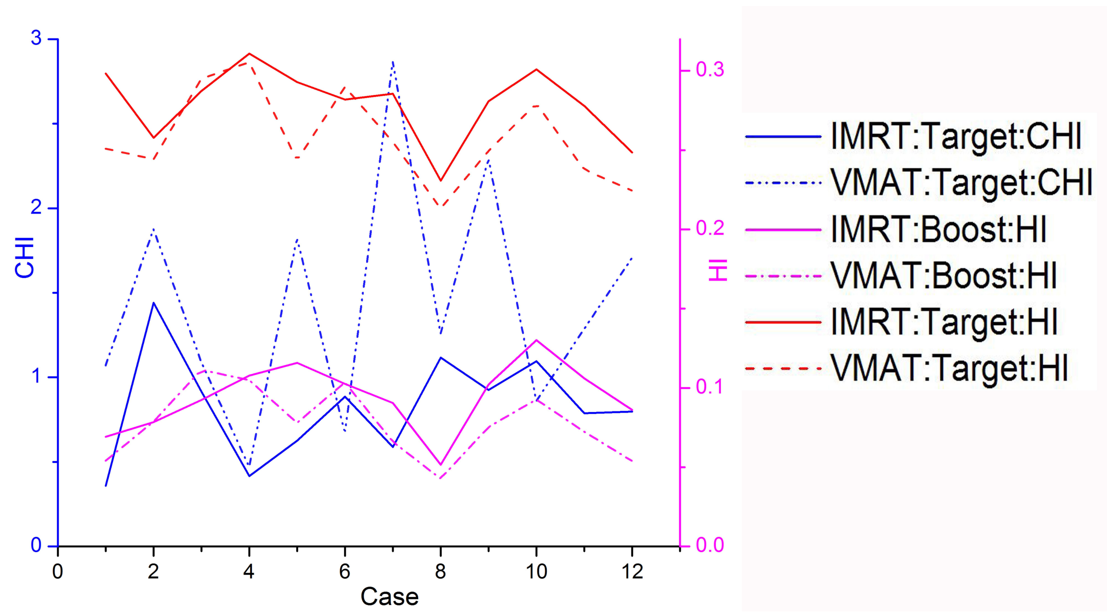

Supplemental Figure 4: HIs of Boost, Target and CHI of Target in two plan techniques for nasopharyngeal cancer.
